# “It’s cleaner, definitely”: Collaborative Process in Audio Production

**DOI:** 10.1007/s10606-022-09448-1

**Published:** 2022-11-14

**Authors:** Thomas Deacon, Patrick Healey, Mathieu Barthet

**Affiliations:** 1 Media and Arts Technology Centre, School of Electronic Engineering and Computer Science, Queen Mary University of London, London, E1 4NS United Kingdom; 2 Cognitive Science Research Group, School of Electronic Engineering and Computer Science, Queen Mary University of London, London, United Kingdom; 3 Centre for Digital Music, School of Electronic Engineering and Computer Science, Queen Mary University of London, London, United Kingdom

**Keywords:** Audio production, Design ethnography, Human interaction, Workflow

## Abstract

Working from vague client instructions, how do audio producers collaborate to diagnose what specifically is wrong with a piece of music, where the problem is and what to do about it? This paper presents a design ethnography that uncovers some of the ways in which two music producers co-ordinate their understanding of complex representations of pieces of music while working together in a studio. Our analysis shows that audio producers constantly make judgements based on audio and visual evidence while working with complex digital tools, which can lead to ambiguity in assessments of issues. We show how multimodal conduct guides the process of work and that complex media objects are integrated as elements of interaction by the music producers. The findings provide an understanding how people currently collaborate when producing audio, to support the design of better tools and systems for collaborative audio production in the future.

## Introduction

Audio producers commonly compose audio content for many sectors of the creative industries, such as commercial pop songs, sound and music design for advertisements, games, and immersive media. However, contemporary audio production workflows are often designed around single user processes and conventions, so how these systems can be used collaboratively is an open, but developing, problem (McGarry et al. 2017). A reason to develop collaborative audio production (CAP) technologies is the evolving landscape of working practices, music distribution, and audience needs. Recently, the COVID-19 crisis has created a seismic change in the needs of every industry to adapt working practices, and professional audio production is no different. Producers are forced to negotiate previously co-located workflow using workarounds and substitutes for activities that happened naturally in the studio. This has led to a renewed interest in CAP technologies as a way to enable producers to work more effectively and efficiently, regardless of their location. In addition to the immediate pressures of the COVID-19 crisis, new consumer technologies often prompt audio producers to adapt and develop new ways of working (Byrne, 2012). One effect of this is the merging of roles in the production chain, meaning more work being done by multi-disciplined, professional audio production workers (performing multiple roles that were previously whole job titles (Kling, 2014)). Another issue is that workflow in the studio is changing with recent developments in (i) networked workspaces for audio production (Fencott and Bryan-Kinns, 2013); (ii) object-based media production workflow (Bleidt et al. 2015); (iii) semantic digital music objects with improved metadata (McGarry et al. 2017); (iv) and spatial audio production for immersive media (Deacon et al. 2019).

Each of these technologies changes how audio is represented and acted on in professional work. But these technical developments do not resolve the basic human interaction issues of collaborating on complex content. Therefore, the design of the interfaces to support new technical architectures needs to be carefully implemented and tested with an awareness of collaboration and domain constraints. This means analysis of media production workflow in professional studios can benefit from, and contribute to, research in Computer-Supported Cooperative Work (CSCW) (McGarry et al. 2017).

The frame we adopt in our research is consider how we can transition from co-located practices to remote ones for professional CAP. But, to do this, we must first understand the situated aspects of human-human interaction (HHI) in a common CAP setting. Aiming at this problem, our paper is a design ethnography study (Crabtree et al. 2012) that uses detailed video analysis to develop an understanding of how audio producers collaborate in their work. One concern in the paper is to develop an understanding of how audio producers collaborate before technologies and new work processes intervene on their skills and methods. Going beyond this, we wish to guide the development of new technologies and processes that make sense for the professional audio production industry. Our analyses in this paper address the exploratory research questions: *What are the current, situated resources for collaborative audio production and how are they used? Also, how do visual, sonic, and social resources interact in a studio setting?* These questions draw attention to coordination features in joint activities, in a co-located setting. Our findings highlight the interplay between resources of situated media (shared screens, control devices, sound playback) and HHI. This provides an understanding how people currently work, in order to design better tools and systems for collaborative audio production in the future.

## Describing Interactions in Studio Audio Production

Professional audio production often involves creating new content based on a client’s brief, personal ideas, or by iterating existing audio content delivered by third parties. Professional producers’ have three distinct skill areas (McGarry et al. 2017; Lefford and Thompson, 2018):

1. Extensive experience of digital audio creation, manipulation, and evaluation.
2. Situated knowledge of working in a studio environment.
3. Practical experience of the content production pipeline.

Skills areas 2 and 3 make professional audio production a social activity that requires communication, planning, composition, content management, instrumental performance, and joint creation of content (Lefford and Thompson, 2018). So, professional music production is a creative process that takes place in a social sphere. When making creative decisions and taking risks, music producers who are working together must decide as a group what to do. For instance, deciding what content is appropriate, judging sonic and stylistic boundaries, and considering external constraints (e.g. from a client). As a social activity, understanding the characteristics of collaboration in music production is necessary to design technological support. In modern audio production, collaboration can occur at many points, such as: (i) jamming in a recording studio (Nabavian and Nick Bryan-Kinns, 2006); (ii) iteratively recording audio to replace existing content (McGarry et al. 2017); (iii) geographically situated songwriters producing content for a variety of projects (Bennett, 2014); (iv) remote bands and artist teams exchanging content over the internet and file servers; or, (v) meticulous crafting of segments of a composition by two (or more) musicians sitting side by side (Brooker and Sharrock, 2016). In this paper we observe features of (v) and offer design implications for the support of (iii), (iv), and (v).

CAP is a rich field of study for CSCW regarding the creative industries because of its mixture of co-located and remote interaction sites, use of digital platforms, and advanced DAW software. However, it is apt to question how the understanding of practices within CAP can contribute to CSCW research. One way to think of such creative collaborations and the products they produce is as part of a distributed cognitive process (Sawyer and DeZutter, 2009). Cognition is distributed among members of the group, through the coordination of internal and external structures (e.g. technology, audio), and in time, as products of earlier events can transform the nature of new events. To produce data suitable to understand a distributed cognitive process, we need to go out into the field and observe behaviour.

Previous fieldwork in CAP research put forward the *Stimulus Evaluation Model*, which features six non-linear and interacting processes - stimulus, approval, adaptation, negotiation, veto and consensus (Bennett, 2012). The ability to generate a suitable stimulus, in relevant media, is the traditionally “musical” part of audio production. But stimulus does not only refer to audible stimuli of music or sound design. Other stimuli can each provide relevant cues for joint musical creativity, for instance language (Nabavian and Nick Bryan-Kinns, 2006), computer interface feedback (Bryan-Kinns, 2013), drawings (Thiebaut and Healey, 2007), posture and gesture (Rahaim, 2008). All these stimuli can be used to structure co-writing. The processes of social evaluation in the *Stimulation Evaluation Model* of musical co-writing (Veto, Consensus, Approval, Adaptation, Negotiation) retain common language meanings but are used with respect to music. Using such a model suggests that exchanging ideas, in response to various stimuli, is a fundamental process of CAP.

But how does *Stimulation Evaluation Model* unfold, based on situational resources such as audio playback? Common ways to interact with collaborators in audio production are speech, gestures, posture, and emotional expression. Critically, music also guides actions and attention by providing a shared context (Brooker and Sharrock, 2016). Viewing work in a music studio as a form of problem solving, we can analyse how producers are *making aesthetic assessments* (Albert and Healey, 2012), both of the music and of each other’s contributions. Making assessments and responding to them is an everyday social action (Goodwin and Goodwin, 1992). Similar to common ground (Clark and Brennan, 1991), the process of arriving at a decision suitable to collaborators is iterative and based on jointly available resources for communication (Heritage and Raymond, 2005). Basic features of conversation utilised in the process of aesthetic assessments include (Albert and Healey, 2012): sequence and turn-taking, preference organisation, epistemic authority, and topical/parameter shifts.

We use Stimulus Evaluation as a guide for our study analysis of language, musical playback, gesture, posture, and computer interfaces (DAWs). Within that analysis we use the idea *making aesthetic assessments* to observe shifts in conversational topics within multimodal interactions that incorporate sound. To demonstrate, consider the following made up example. Imagine two audio producers sitting side-by-side in front of a DAW screen discussing a drum loop:

- 1 A: that sounds busy to me
- 2 B: yeah, but it's energetic

In the simple exchange above, *A’s* ‘busy’ assessment might imply that the drum loop is messy. Following this, *B’s* assessment acknowledges *A’s*, however, *B* shifts consideration onto an aesthetic attribute of the drum loop, ‘it’s energetic’. This toy example shows that, in certain circumstances, judgements about aesthetics can be based on the systematic differentiation of people’s opinions on assessments (Albert and Healey, 2012). Understanding music producers’ aesthetic assessments of stimuli guides us onto features of collaborative workflow that are important to group members.

## Research Approach

The goal of this study is to support future research by understanding how technology is used and situated within social interaction. Design ethnography is a suitable method for developing a detailed understanding of how people interact in a studio setting (Crabtree et al. 2012). The approach to fieldwork and data collection was based on a passive participation stance. This involved shadowing, recording videos, some discussion during the process, and conducting interviews on-site. This approach can access tacit actions and language that forms professional audio work (McGarry et al. 2017). The outcome of the research is a set of concepts and models that can sensitise design to important features of social interaction related to technology usage (Hammersley, 1992). Our approach supports new technology research by highlighting how to design systems compatible with the everyday lives and skills of audio producers.

### Setting - Who, What, When, Where, and How

**Fig. 1:**
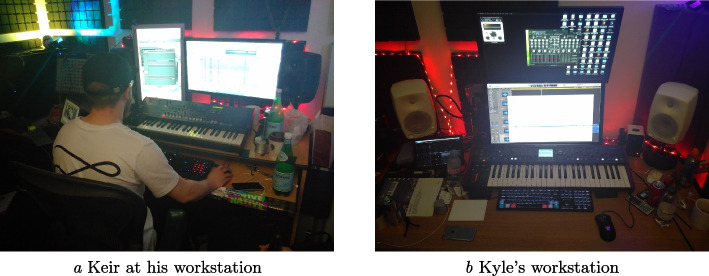
Commands studio workspace

**Fig. 2:**
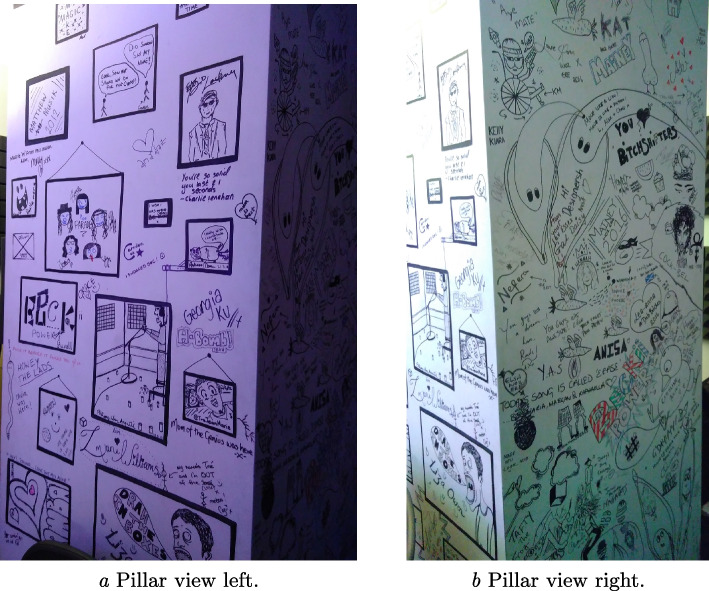
Commands studio pillar views, inscriptions from Commands and previous session artist collaborators

Fieldwork was conducted at the workplace of *Commands* (Kyle & Keir), a versatile and much in demand production duo based in a professional music studio in central London. *Commands* work regularly with international pop artists and record companies. The duo has extensive, individual and shared, professional production experience in electronic dance and pop music. Both are trained musicians and producers, and have experience of performing in bands and as solo artists. The fieldwork took place over 3 months, with five sessions worth of video, along with a series of other visits on non-consecutive days. The musical content being worked on each visit was different. The basement studio is situated with six other studios belonging to their publisher, a studio manager’s office, a shared kitchen and toilets, and a stairwell up to the outside world. The studio is a room cut off from the outside world by a heavy, soundproofed door. The room contains a vast array of audio production equipment and musical instruments; from simple toys through to expensive professional ‘gear’. The room is fitted with a mixture of acoustical padding material and various LED lights. Views of their workspaces can be seen in Figure 1. While being a technical space with specific perceptual features for music production, it is also a social one, people drop by from other studios in the building to chat, collaborate, and introduce clients. This structure provides access to new collaborations with artists or record companies. Figure 2 highlights a structural pillar within their studio, that acts as a situated history of this social interaction with numerous markings from visitors. The workspace and collaboration style of *Commands* is common for their industry, with writing teams often being co-located geographically (Bennett, 2014).

*Commands’* work covers a range of activities: (i) recording sessions with vocalists; (ii) writing, production and mixing of music for other artists, both ghostwriting^1^ and production as the *Commands* brand; (iii) remixing of songs; (iv) production of samples and tracks for library music; (v) production of music and sound design for moving image; (vi) making of music for individual artistic projects with other collaborators. Work activities not currently undertaken by *Commands* include (i) full band or instrumental recording sessions; (ii) mastering of songs or albums; (iii) preparation of music for live performance.

The study was carried out following ethical review by the Queen Mary Ethics Committee and all participants provided informed consent and agreed for data samples to be shared (Approval ID QMREC1619).

### Data Analysis

For the analysis in this paper, phases of work are articulated by their interactional features. Detailing interactional features focuses analysis on the real-time performance of activities, situated in a shared social and material space, using video-based interaction analysis (IA) (Jordan and Austin Henderson, 1995). Aspects of the IA were discussed in collaborative data sessions (Albert, 2017). The sessions focused on short segments of video data where the *Commands* duo works together on a song. The data format used in the presentation did not include any inferences collected during the analysis and coding, in order to have themes surface in the collaborative data sessions themselves. The findings and discussions of data sessions have been integrated into this work.

For the presentation of data, transcriptions include simple turns of talk, gesture, gaze, and posture. Adapting a heuristic device from previous research, transcriptions include references to audio playback and interface interaction during phases of action (Brooker and Sharrock, 2016). The combined transcription of interaction aims to address how sense-making is socially distributed and structured around the playback of audio and interface use. Fragment I is an example of the transcription method.

#### Fragment I: Fragment example where the pair decide what files to deliver to a client.


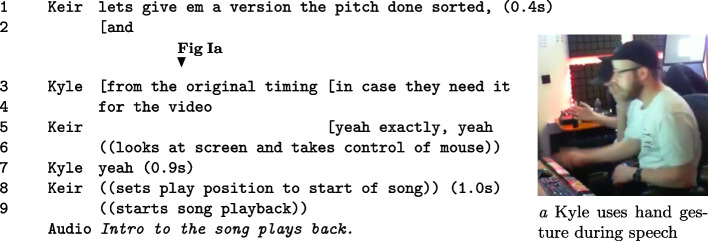
 Each numbered line indicates a turn of speech or action. Within single brackets, gaps in speech and action are marked in seconds e.g. lines 1, 7, and 8 (L. 1,7,8). Physical or digital actions are indicated in double brackets (L. 6,8,9). Single opening square brackets line-up turns, to indicate parallel speech or action (L. 2-3,3-5). Unnumbered lines indicate either turns of *Audio* or figure references. Audio turns describe audio playback and sonic qualities. Figure references indicate the sequential timing of a reference e.g. above line 3 referencing Figure Ia.

### How is Audio Produced and How Does a DAW Work?

**Fig. 3:**
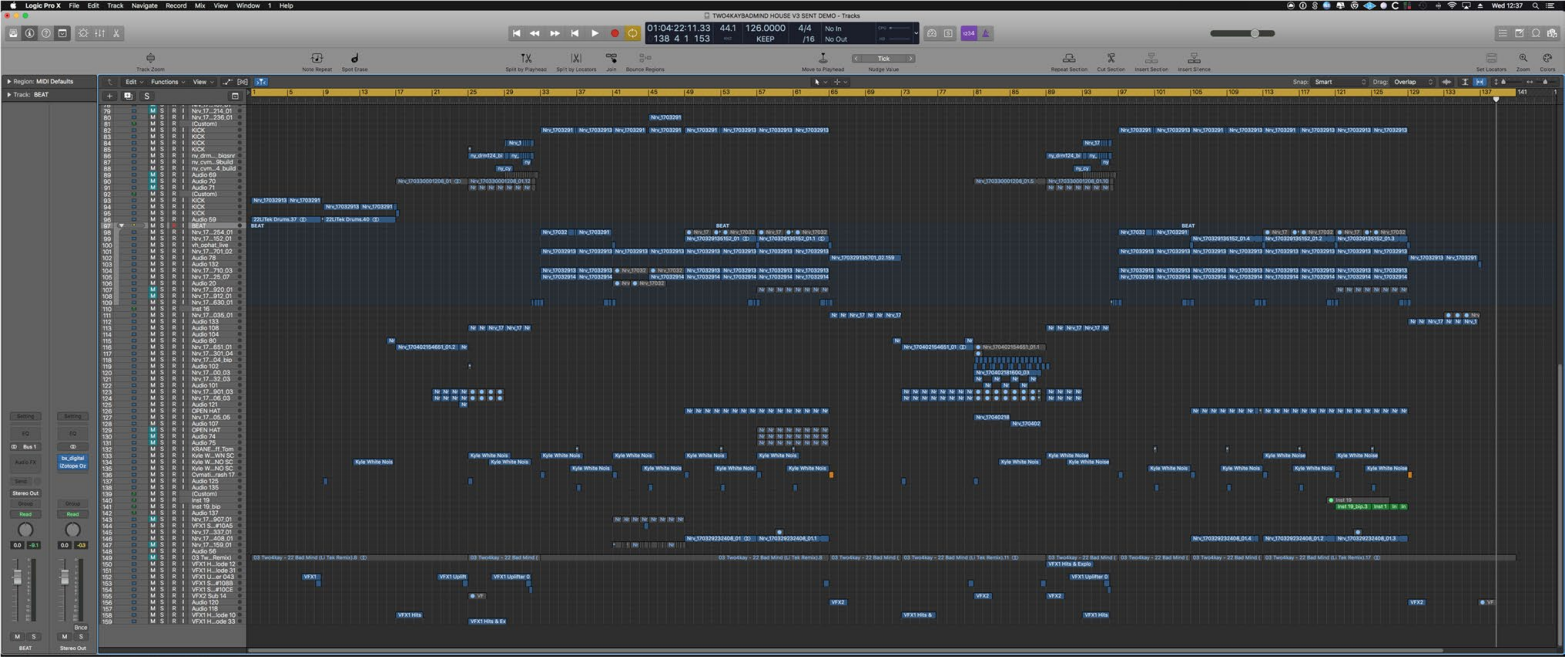
Example of *Commands* audio content arrangement in the Logic DAW timeline. Song is one of the versions used in the analyses of Section 4.2

For those unfamiliar with audio production, this section provides a short review of key workflows in audio production using DAWs. *Tracking* is the live recording of audio into a DAW. *Sampling* is the use of pre-made audio files to produce layers of instrumentation. *Synthesis* is the use of sound generators to produce instrument layers for a song, synthesis is often MIDI^2^ controlled. *Editing* is commonly associated with temporal selection, cropping, and arrangement of audio content in a DAW *timeline*, see Figure 3 for example. *Mixing* is the balancing the levels and frequencies of different parts of a song. In practice, a mixture of simple and advanced signal processing strategies are undertaken to mix a song. Within a DAW, whole workflows with dedicated UI views are used for mixing. *Rendering/bouncing* audio is the term used for the exporting audio files from a project, often for archival or distribution. Associated with bouncing out audio is the term *Stems*, these are often stereo recordings of parts of a song combining multiple individual tracks. For example, a drum stem will typically be a stereo audio file that sounds like all of the drum parts mixed together.

Some other practical terminology includes: *Master track*: is the main audio output for all audio tracks in a DAW session. *Solo/Mute*: Soloing a track means listening to it by itself, in DAWs multiple tracks can be soloed simultaneously, muting is silencing a track. *Bussing*: mixer routing abstraction process where the outputs of a series of tracks are routed together to apply group-level effects processing and volume control. *Equalisation (EQ)*: amplifying or attenuating the levels of different frequencies in a signal. *dB*: stands for decibel, the unit of sound intensity; in audio production, it is used for mixer meters and volume control amounts. *Side-chaining*: a form of audio signal cross-modulation used to change the volume (or dynamic range) of a track based on another; commonly used in mixing to balance elements creating ‘space’ in the mix. *Clipping*: is when an audio signal is amplified past the maximum allowed limit, it leads to distortion and a lowering of audio quality. *Stereo Imaging*: is the positioning of sounds using two outputs (speakers or headphones), it is also used to create a sense of space for the listener.

## Case Study Observations and Findings

Section 4.1 describes broad trends in *Commands’* workflow based on analysis across the whole ethnographic process. Section 4.2 presents features from one day, where segments from one song were worked on, the observations are accounts of how people work together to create audio content.

### Organisation of Project Resources in the Chain of Production

This section presents an overview of how *Commands* structured their work processes for music production. Audio production work requires high levels of detailed editing in complex project hierarchies. This makes project and asset management a key concern from the outset of new commissions. DAWs have abstractions for managing sessions and file variations within them, but in their workflow, *Commands* manage numerous file and folder structures explicitly; this is similar to previous research findings (Duignan et al. 2010). The following wordings are based on *Commands’* language and organisation process, rather than software or industry terms.

**Commission**: A collection of work that can contain multiple different songs to be completed. Examples include producing a series of songs for an album or single release. All the commissions worked on during the ethnography had between 5 and 15 stakeholders. Management of the chain of production is distributed across many versions of projects across stakeholders.

**Fig. 4:**
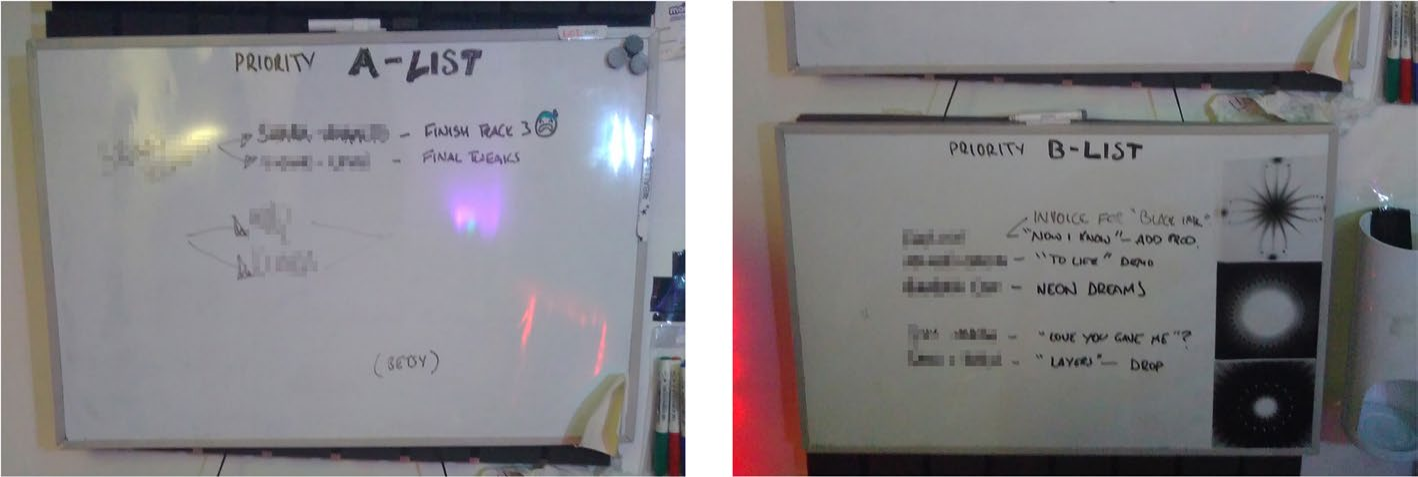
Whiteboards used by audio producers to indicate active projects, and their priority for delivery. Client names have been pixelated

**Project**: An individual song within a commission. In the case of remixes, the projects are kept separately. As data, the project level is exemplified by folder structures for each project part. These link together with email communications and a whiteboard in the room that indicates different projects’ priority (Figure 4).

**Version**: A part of a project that contains aspects of the song, feeding toward the final submission of the project. Versions are key communication points with clients. Versions form branch points for the duo to organise content variations that can be easily referenced and delivered to clients e.g. v1, v2. This is primarily used in conjunction with version-specific naming e.g. “Drum Fix”, or “Pre-Mix”. This stage of organisation exists at the folder level managed by the pair on individual machines, cloud delivery services, and their studio network storage. Each version is represented as data by stereo bounces, bounced stems, or DAW project files.

**Sessions**: Within different versions of a project the duo will conduct work on sessions, such as drum loop production or vocal mixing sessions. These branches allow the pair to work on different aspects of the track synchronously without needing to be on the same machine. This is the level of DAW project files, but for archival purposes sessions may exist as stems without project files.

**Tracks**: Each session is made up of tracks e.g. vocal, guitar, drums, and sub-mixes. This term is used contextually to mean different things by the pair. For instance, a track can mean versions or project. More concretely in the production process, a track is a discrete component of audio, it can be either mono or stereo recorded audio or a MIDI instrument. Hereafter, the usage of the term Track refers to the concrete production process meaning. Individual sessions (DAW files) count between 10–300 tracks. The way *Commands* work means final versions typically have between 100-200 individual tracks, often with complex sonic interdependencies in the structure of each version.

### Interaction Analysis of Collaborative Audio Production with Digital Audio Workstations

In the following fragments, a dancehall/afrobeat song, composed and produced by *Commands*, has been returned to them with notes on ‘things to fix’ based on artist comments and the commissioner’s recommendations. In that context, the following phases of work are analysed:

- Feature A: Finding the Problem - to move forward with work, the pair collect project resources and sound materials to relate to client instructions.
- Feature B: Fixing the Bass - a breakdown of *Commands’* decision process for what action needs to be taken on the bass instrument.

Within the two features we analyse the process of making aesthetic assessments. This focuses in on the process of creating digital music objects, by looking at how joint attention and mutual understanding are negotiated during work. To set the scene, we highlight that all work reviewed in fieldwork was client projects, where a client briefed *Commands* with a concept, some reference materials, a skeleton track, or instructions for revisions to a previously delivered piece of work. However, client instructions were often quite vague. This means the overall direction of work is known, but there is still work for them to interpret instructions.

**Fig. 5:**
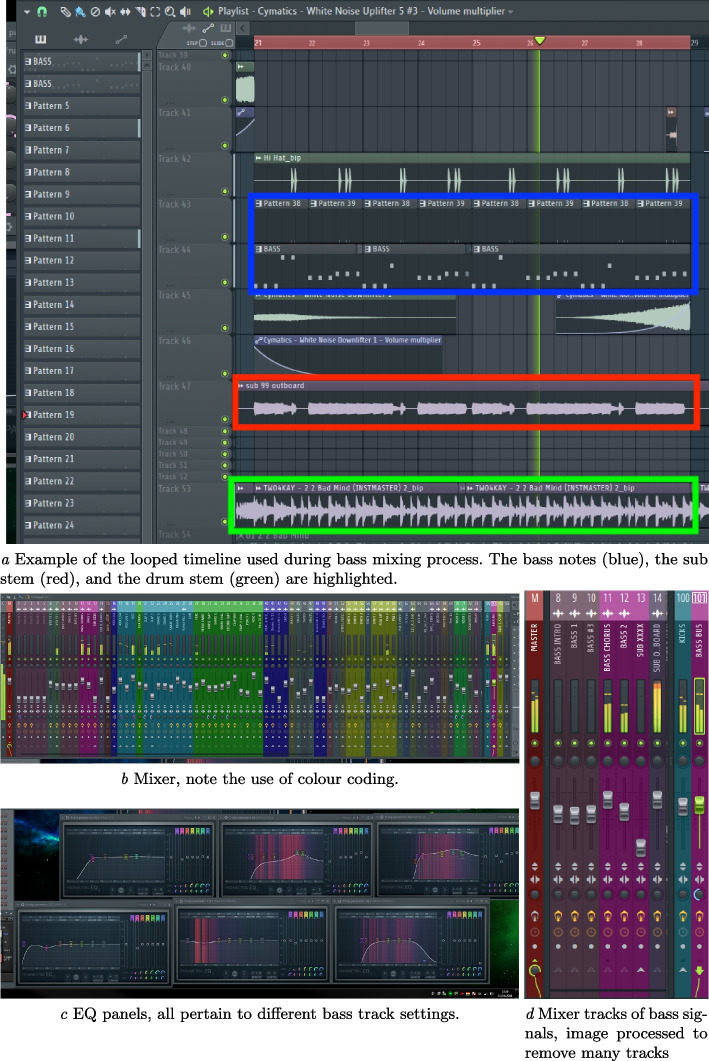
DAW views used during the bass work

In both features, *Commands* work on the bass sound of the song using the session views shown in Figure 5. The bass sound is made up by seven individual tracks and a *bass buss*, these can be seen in Figure 5d, the track number, *names*, routing, and functions are as follows:

- 8  *Bass Intro* - 101 - intro, pre-chorus, and verse sound
- 9  *Bass 1* - 101 - intro, pre-chorus, and verse sound
- 10  *Bass #3* - 101 - intro, pre-chorus, and verse sound
- 11  *Bass Chorus* - 101 - chorus sound
- 12  *Bass 2* - 101 - chorus sound
- 13  *Sub XXXX* - Master - unused legacy sound/track
- 14  *Sub Outboard* - Master - main sub
- 101  *Bass Bus* - Master - group buss

The ‘sub’ is a bass sound, it refers to frequency components < 100Hz. Each mixer track of the bass sound has an EQ, used to sculpt the sonic profile of that sound; these panels can be seen in Figure 5c.

#### Feature A: Finding the Problem

During audio production, there is a need to establish mutual understanding in a work session. But, this can be a difficult process given the complex nature of the work. The analysis in this section breaks down one instance of how *Commands* set their focus on work-to-be-done in the DAW. For context, both participants sit side-by-side at Keir’s workstation, with Keir in control of the DAW, while Kyle spectates and occasionally checks his mobile phone. Before this, preparation work included informal discussion while sourcing files, opening project versions in DAWs, and checking emails. The task in this phase of work is deciding what things they are doing to the bass part, concerning client notes. The changes to be made at this point are unknown beyond that the bass part needs to be ‘fixed’. This could be done in many ways, but it focuses the pair on parts of the music arrangement that make up the bass instrument and what it interacts with.

The action in Fragment II highlights a negotiation of what the problem is, and how they should be focusing efforts to move forward. This requires each of the pair to present their reasoning of what the problem is in the mix, in order to progress with work. In the fragment, the following language is used: ‘kick’ refers to a bass drum sound; ‘Scooped’ is a reference to an EQ technique where the lower frequencies of a signal are removed; ‘Peak’ refers to a signal that is too loud so it’s visual indicator ‘peaks’ at a specific moment in time; ‘Pre’ means pre-chorus, a song structure component.

##### Fragment II: Diagnosing issues with the bass instrument. In Fragment Figures IIa-IIc, Keir (right) and Kyle (left) use gestural displays to draw attention to different features of the DAW mixer shown in Figures 5d and 5b.


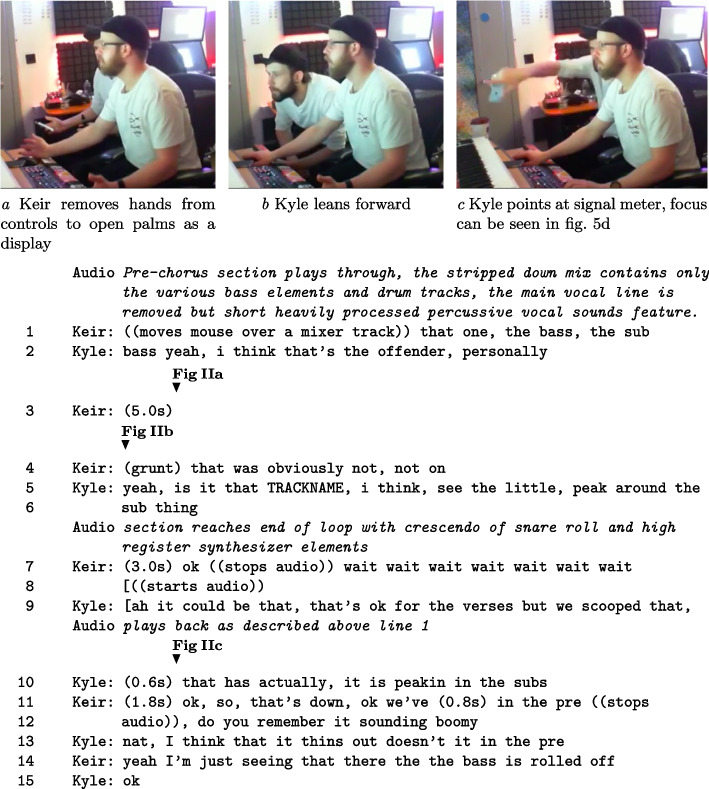
 In Fragment II, a form of distributed memory recall is witnessed with Keir’s question about whether the bass sounded “boomy” in the pre-chorus. Memory is distributed as Keir has some of the information needed, but utilises Kyle’s knowledge of the song structure through a recall request. It highlights the contextual relationship of sound to production practices in the studio; existing research would describe this as a form of situated metadata (McGarry et al. 2017). For this situation, targeting changes means understanding which version was heard by the client, but also how they made changes to the content. For studio-based CAP, this demonstrates a reliance on multimodal (speech, gesture, computer display) referencing to construct common ground. As they investigate the bass audio features in Fragment II, different problem sources are proposed such as the tonal balance (“boomy”) and processing choices (“scooped”). The fragment action highlights how the pair aligned each other’s focus. The joint attention process interleaves multiple project files, sonic properties, live playback of track content, and visual representation, all in real time. We describe this phase of joint problem discovery as *attunement*. Upon proposing what an issue is, the collaborative effort is on making sure each other is considering the same problem concerning the complex resources and, possibly, ambiguous instructions. This phase of action highlights how joint attention is necessary to establish what work needs to be done in complex projects.

#### Feature B: Fixing the Bass

Continuing directly from Feature A, the next series of fragments focuses on a process of structured experimentation. In the action, *Commands* negotiated how to proceed with corrective actions on the bass elements. Previously, they had isolated that the ‘sub’ may cause problems for the overall mix by being audibly distorted (‘clipping’). In this section, *Commands* query details about the bass assembly, suggest amendments, and trial equalisation settings across time. They attend to a looped section of the song attending to the timeline (Figure 5a), the mixer (Fragment Figure 5b), and effects panels (Figure VI). Throughout the phase, suggestions are offered and actions are taken to explore problem areas, where the pair utilise a variety of interactional resources to establish common ground. In audio terms, processes in the following fragments include:

- Discovering the routing of bass parts in the virtual mixer;
- Reducing volume on individual bass parts and the buss;
- Applying equalisation (EQ) to bass parts;
- Checking changes using solo and mute functionality.

##### Finding the right problem

The next two fragments demonstrate how embodied action influences stimulus evaluation processes. Fragment III shows how Kyle tried to get Keir to listen to him and use his proposed EQ solution. Due to asymmetric control opportunities, Kyle cannot change items in the DAW. Immediately before this action, the pair had determined that the sub-track does not get processed along with the other bass parts in the ‘bass buss’.

##### Fragment III: Offering suggestions for content actions. Figures IIIa-IIIc indicate bids for attention using gesture; Kyle on the left, Keir on the right.


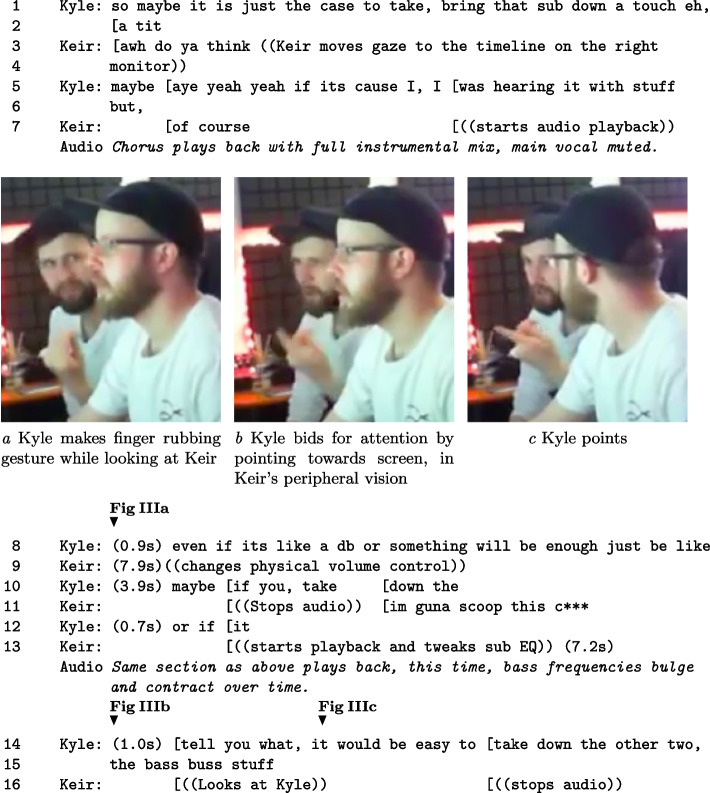
 In the action of Fragment III, Kyle attempts to secure Keir’s attention through gestural interaction and speech during audio playback. The pointing gestures used are interspersed with Kyle’s description of why his solution is warranted. Recalling the literature on making aesthetic assessments, the sequence of action highlights an attempt by Kyle to alter the information territory of the content action, by articulating what should happen. This can imply a level of ownership by Kyle in how to evaluate the matter assessed (Heritage and Raymond, 2005). He does this by stating the parameters that should be used to investigate the issue. Examples are taking the volume down on ‘that sub’ and ‘the bass buss stuff’, instead of Keir’s strategy to make EQ changes. This also occurs through a self-repetition of advice to take the volume ‘down’ (L. 1,8,14). This trading of territory is necessary for collaborative progress rather than just Kyle watching what Keir does. As Keir manages relative epistemic rights to evaluate states of affairs, by controlling actions in the DAW, Kyle is required to use bids for attention to state what assessment is of importance. If Keir ignores Kyle’s requests, it stops being a collaborative situation. While not unique to CAP, embodied action is a useful coordination resource in the studio environment as: (i) the music is playing and being concentrated on; (ii) Keir’s visual attention is focused on the screen as he exercises his plan of action. Later, Keir actions Kyle’s request, to mutual satisfaction.

Fragment IV highlights the use of gaze, posture and referencing to make assessments on the current state of the bass assembly. This fragment highlights an array of referencing actions by Keir that are interleaved with talk, while Kyle states his assessment of the problem. In the text, *global volume* refers to the output level of the speakers controlled via a hardware volume controller.

##### Fragment IV: Referencing and quality assessment.


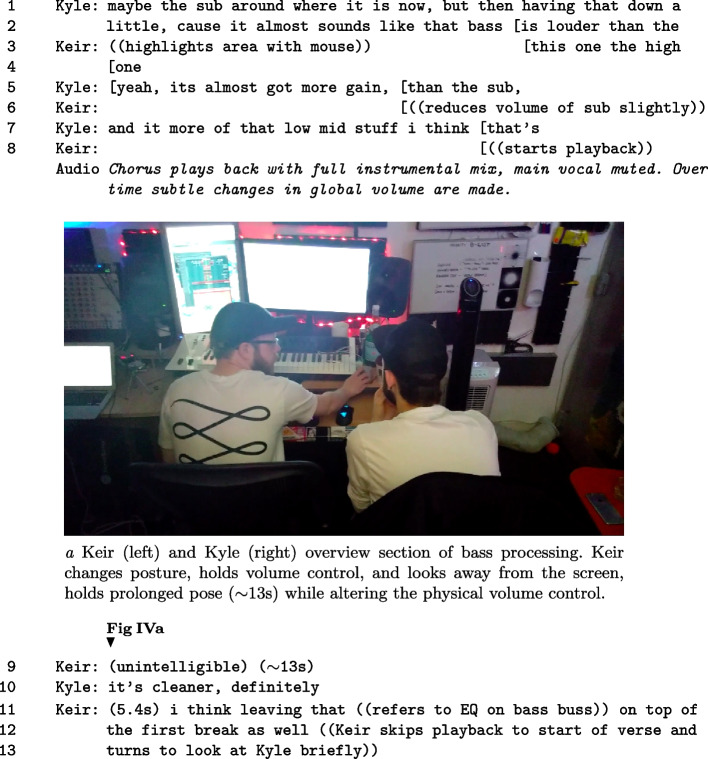
 In a specific feature, Keir alters posture to maintain gaze and hold the physical volume control knob for an extended period $({\sim }$13s), seen in Figure IVa. This prolonged gesture is ambiguous. In terms of control, the volume level was altered in the first seconds of action, and to a tiny amount. The remainder of the posture change can be interpreted in many ways: waiting for a reply, focusing himself or Kyle on the mental act of listening, or being ready to make another level change related to critical listening. Previously mentioned, Kyle’s suggestion of solutions through this period were selected and carried forward, to mutual satisfaction. Keir controlled the implementation of solutions, proceeding with his plans, ignoring Kyle’s initial suggestions. This is not excessively negative. We must proceed through our own understanding of the problem, but Kyle’s repeated mentions of a simple solution were not acknowledged until he guided Keir’s attention using an array of multimodal conduct. These last two fragments show how physical gestures can be designed to focus on listening, which provides insight into the needs of CAP.

##### Final Changes

The following fragments show how Commands listen to content in different ways, such practices help the pair to make decisions about the mix. In Fragment V, Keir operates the DAW, while Kyle spectates, and the pair isolate elements of the song to evaluate mix changes. They play a segment of the song on loop while discussing sonic and technical characteristics.

##### Fragment V: Discussing processing: Kyle states that the bass sound should be a prominent feature of the song’s sound.


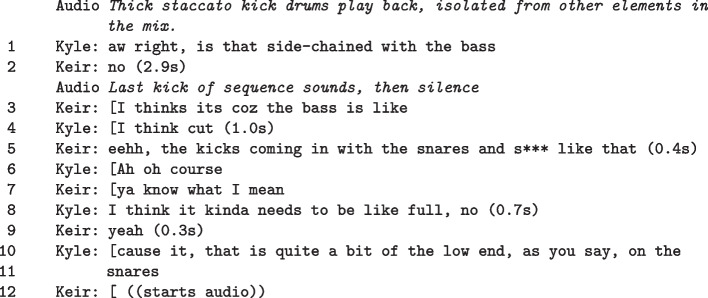
 In this action Kyle positions an aesthetic assessment of what the quality of the audio should be (L. 8). The position of this assessment in dialogue creates a parameter shift in negotiation, suggesting that the problem should be resolved on a sonic target for that instrument (a bass being made to sound ‘full’ would be different to making a vocal sound ‘full’, because they occupy different sonic space). Kyle’s assessment that the bass assembly must sound ‘full’ is developed further in the action of Fragment VI, where Kyle asserts that the current state of the sound matches previous assessment of ‘full’. In the action, they intersperse listening with discussion while changes are made to mixer settings and effects processing.

##### Fragment VI: Setting levels


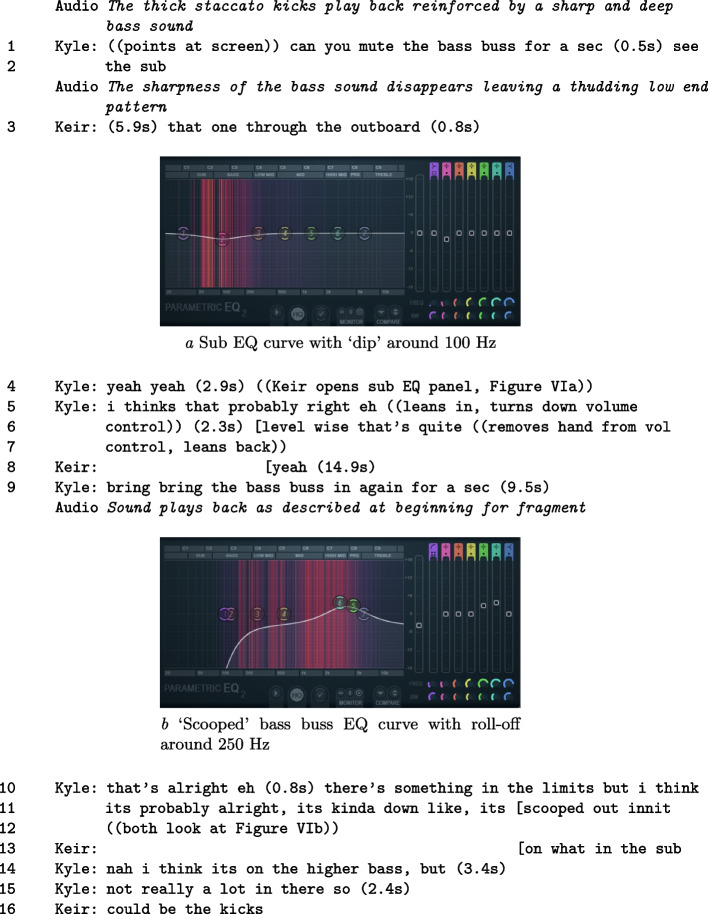


In the action, Kyle sets constraints on the sonic problem space by asking Keir to mute the bass buss (L. 1). He does this to explore content relationships. What happens in the subsequent turns is a series of topical parameter shifts, based on new assessments. Those assessments are made possible by changing the perceptual structure of the problem. To improve critical listening capacity, Kyle leans forward (L. 5), adjusts his head position (changing his stereo image), and adjusts the global volume. Changing head position in relation to the speakers alongside volume, alters his perceptual thresholds of sound relationships in the signal^3^, allowing an alternative way to hear issues and combinations. These changes to perception are consolidated by a period of focused, critical listening. Again this highlights how gestures are designed to support the process of listening. In the flow of actions, further modifications to the problem space are made by taking out and putting back in various instrumental elements. Also, on-screen information about the bass buss signal and effects processing (L. 11, Figure Vb) are woven into technical and aesthetic assessments on the problem. Despite not having control of the DAW, the current territory of assessment is being guided by Kyle. To make these assessments, he uses interactional, perceptual and material resources. The final result is a more clarified understanding of what the problem is and means to remedy it supported by updated information from Keir.

To summarise, as work progresses the sonic, representational, and linguistic state of bass elements are queried and reformulated multiple times. Partitioning of sonic space, is achieved by changing audio settings (switching in and out mixer tracks, effects processors or the global volume control) and posture. This *experimentation* allows the pair to alter perspectives on the underlying audio materials, iteratively focus on sonic problems, and assess goal structures in terms of stylistic considerations.

## Discussion

Throughout the fragments, a series of multimodal conduct were observed. Our findings highlight the situated resources used in this creative collaboration. We discuss observations in the following sections:

- *The Multimodal Conduct of Decisions* - Collects the instances of interactional resources used in fragments and describes their functions.
- *Collaborative Audio Production Interactional Practices* - We extend previous analytic categories and propose other features of social interaction in CAP.
- *Interplay of Visual and Sonic Complexity* - Here we integrate analyses to propose a key design focus.

Also, the boundaries of the study and methodological issues are discussed in the *Caveats* section. Hereafter, fragment references are indicated in parenthesis as (F. x).

### The Multimodal Conduct of Decisions

DAW-based collaborative audio production is an interactionally-dependent process, where aesthetic decisions are embedded in social practice. In our data, content assessments were often marshalled using embodied behaviour (F. II-IV). *Commands* utilised an array of multimodal resources to move forward with work and support mutual understanding of problems. Multimodal resources include:

- Specific audition of audio related to work tasks (all fragments).
- Referencing static and temporally fluctuating on-screen representations of audio using gesture (F. II).
- Referencing on-screen representations of audio and effects processes using deictic and named references (F. II, IV).
- Recalling distributed memory of project audio processes (F. II).
- Gestural bids for attention in the process of work (F. III).
- Posture changes (F. IV).
- Highlighting areas of interest on-screen with the mouse pointer (F. IV).
- Shifts in gaze from screen to mutual gaze (F. III-IV).

The contribution of gesture to the co-ordination of audio production cannot be underestimated. The availability of information about a collaborators’ gestural and postural relationships concerning specific references, timed precisely, is of importance. This is because embodied conduct in the studio structures how people problem-solve sonic issues. To diagnose problems, *Commands*: isolated sources and made available their assessment (F. II), coordinated media (F. IV), and directed action (F. VI). Gesture, posture, gaze, and speech, each offer ways to separate features within the shared problem context. For instance, *Commands* guided each other’s attention to facets of the sound and previous assessments while downgrading or upgrading specific assessments made in the process (F. V-VI). Each participant had to highlight the merits of individual solutions, often after trialling failed strategies. Here, interactional work supported the refocusing of efforts on other candidate solutions. To consider a solution, requests for reasoning create a requirement for an individual to explicate their current strategy. Similar to the appraisal of static visual art (Heath and Vom Lehn, 2004), a function of embodied conduct in making assessments is to continually establish co-orientation towards particular features of sonic objects and encourage each other to appreciate them in specific ways. Precisely what aspects of the song are targeted for assessment vary, but they are reflexively tied to the strategies and perspectives of the speakers assessing them.

### Collaborative Audio Production Interactional Practices

By extending previous analytic categories (Brooker and Sharrock, 2016), we integrate our analyses in the idea of *collaborative audio production interactional practices* (CAPIP), categories include:

**Critical listening**: Focused, collaborative listening of sound to determine problems and elements to develop (F. II-VI).
**Talking about musical ideas**^4^: Deciding what ideas to implement via the software and instruments, these can be new ideas or solutions to problems (F. III-VI).
**Enacting musical ideas**^4^: The work of inputting and refining ideas to achieve desired effects (F. III & VI).
**Reviewing musical choices**: Deciding if enacted musical ideas fit within project constraints (F. II, IV & V).

We acknowledge that these features provide an ordered gloss not present in reality, meaning that within any instant of interaction, multiple aspects of these features could be unfolding simultaneously. We discuss these practices further through three features of social interaction: *Attunement*, *Experimentation*, and *Making Assessments*.

**Attunement** is the uncovering of processes required to progress tasks. It is closely related to the critical listening and reviewing musical choices. In our data, attunement includes the shared analysis of the audio and negotiation of possible solutions (F. II), where *Commands* utilise audio, visual, and distributed memory resources (human and machine). Interaction modalities help to ground information in a shared context (Clark and Brennan, 1991), building levels of attunement to the group’s goals. Key to the attunement phase is that the work of problem solving is mutually resolved. We can see this in transition of problems from high level instructions down to smaller problems, solvable by content actions. Here, collaborators resolve different perspectives on the state of affairs, changing the navigation of the problem space from what might occur in an individual’s process. For example in Fragment II, Kyle interjected on Keir’s process of content action to suggest a simpler course of action to fix the bass instrument.

**Experimentation** links closely with *Talking about musical ideas* and *Enacting musical ideas*. It is the coordination of participation woven into audio production processes. When making content, iterative reflections are based on changes made to sound and other information. This makes experimentation in CAP is form of joint stimulus evaluation. Participants need to resolve different assessments of issues, using multiple information sources such as the audio, the DAW, and shared history of discussion. For instance, in Fragment IV there is an iterative process of collaborative listening, content review, and basic mixing actions (solo, mute, EQ changes). In that process, making clear assessments is complicated by the fluctuating sonic characteristics and multiple control opportunities present in DAWs (e.g. the mixer and EQ in F. VI). This is because it is hard to focus, critically listen, and specifically reference issues when content can change so quickly. This idea is discussed further in the next section.

**Making Assessments** highlights the exchange of information territory in *Commands’* interactions. The territory comprises: parameter shifts in assessments of technical and aesthetic issues (F. IV, V, VI), self-repetition (F. III), clarification requests (F. IV), and confirmation checks (F. IV). The function of territory exchange is to anchor aesthetic or technical assessments to proceed with work. Making assessments is a key behaviour that weaves through all aspects of CAPIP.

### Interplay of Visual and Sonic Complexity

Thus far, we have provided descriptions of what *Commands* did, and given labels for behaviours and practices, but this does not necessarily furnish us with understanding of what is needed to change CAP for the better. It is clear CAP presents many complex challenges for design. We propose that understanding how visual, sonic, and social resources interact is key to designing new systems and support.

Simple, situated, spatial practices allow *Commands* to structure the complexity of the workflow. For instance:

- **Physical/Digital workspaces organisation**: a dual monitor setup is used to spread out pieces of the DAW interface (Figure 1), such as placing the timeline view on one screen and the mixer view on another. This spatial partitioning also gives off signals based on where a person’s gaze is located.
- **Colour coding**: within DAW sessions, colour coding of tracks in a session was a ubiquitous feature of *Commands*’ session organisation; see Figure 5b for an example.

However, project complexity, the temporality of audio, and the need for specific referencing create a tension for professional audio content production. For instance, the auditioning of material features prominently in our data, with subtle sonic changes made in-the-loop. But auditioning is also a task practicality that can structure social behaviour (F. II-IV,VI). This basic process allows continual unpacking of sonic problems. We suggest that the extended temporality created by auditioning underpins the structure of assessment making. To formulate assessments, one has to listen to changes, monitor visual feedback, and socially interact to state claims. Over the fragment turns, significant periods of delay can be seen between utterances (e.g. F. VI L. 8). While somewhat obvious – when producers make music they have to listen to it – the unfolding of sound in time puts a burden on how producers evaluate it collaboratively. Sound happens in time, and this requires *Commands* to point specifically when in time things happen that may be relevant. Also, to make assessments, producers must sample enough evidence to position a claim in the collective information territory, otherwise they must develop a suitable topical shift that is contextually relevant.

The iterative process of contextualisation using the audio and visual resources, mediated by interface control is a key characteristic of working with complex song structures in modern music production. The assembly of on-screen resources (panels for MIDI notes, audio, corresponding mixer channels, and signal processing panel views) are used for focusing attention and directing the action. But temporal variability highlights a problem in the song as a collaborative object, certain features have a level of indeterminacy. The visual representation of music objects exists at multiple levels, some temporally fluctuating (signal meters) and others ostensibly static (timeline representations, effects processing lists). Closely coupled with the audio-visual representations are the interactive features of music objects (equalisation, mixer controls, effects parameters), these elements of sound control can be utilised to continually modulate the other two forms of perceptual availability (audio, vision). What this interaction of representations and control opportunities creates is a level of indeterminacy, as soon as a feature of perception may be grasped in the auditory domain, it changes due to actions in the DAW. Also, this lack of stability is not isolated to one member in a collaboration, each person in a situation may be operating at different levels of critical listening or analytic focus. This social indeterminacy makes referential activity potentially ambiguous and open to interpretation in the process of making assessments.

So, the nature of working with audio can be viewed in a perceptually dependent cognitive loop, that exhibits levels of indeterminacy. But, changing perspective on a sonic problem allows alternate ideas and appraisals to be formed. This is conceptually similar to drawing, writing or other representation-based design problems; collaborators think through, and about, the different assemblages of resources individually and collaboratively. This malleability of what is happening, what could be done, and what should be done to content, provides an important concept for understanding the design of collaborative features for complex media workflows. Following this idea, an alternative way to think about studio work, and potential remote needs, is to consider how information sources are made available. Features would need to allow users to make their understanding available and contribute to their collaborators’ analysis of the materials.

### Caveats

*Commands* usually work on different deadlines, checking in with each other at key points before sending a project back to the client. Also, while the workspace and collaboration style of *Commands* is common for their industry, the solo producer is the most common form of employment in the production chain. What is shared by solo producers and collaborative groups like *Commands* is that content passes through a series of stages (a chain of production). Given this, there are important aspects of work not covered in this study. This study does not address the needs of the workplace that are coordinated at a larger scale, with distributed, temporally asynchronous tools. For instance, developing musical concepts at a very early stage in remote artist collaborations, the transition of projects between multiple parties, and chasing up of payment through a variety of social technologies. The incremental work done in these stages also requires significant collaboration over shared materials. To situate design implications for collaborative systems in the wider context of CAP would also be worthwhile, but doing so would require configuring goals and data collection differently to this study.

The musical work conducted by *Commands* during the ethnography was almost completely done within the DAW or relating to it. Periods of instrumental input (guitar, keyboards, synthesizer) iterated existing content or were used for problem-solving musical issues (harmony finding on a piano). Also the music style covered in our data (DAW-based electronic dance) does not represent all the aesthetic needs of CAP, such as instrumental artists performing, recording, and producing content over a series of days (Nabavian and Nick Bryan-Kinns, 2006; McGarry et al. 2017; Lefford and Thompson, 2018). Also, in their daily tasks *Commands* would switch in and out equipment (e.g. keyboards, guitars, microphones) based on the needs of the job. This is primarily a function of the size of the studio, and the variety of work they must complete on irregular schedules. Also, external hardware for effects processing, ‘out-board’, was used frequently to augment sonic characteristics of tracks in a session. Using the wording of previous research, the use of non-computer based systems introduces contextual meta-data about choices made in the production process (McGarry et al. 2017). But, the study presented in this paper does not extend understanding about how to capture or represent this meta-data in digital objects.

As described in the *Research Approach*, this paper describes the process of music making through interactional competencies with the lens of problem-solving. The paper’s findings analyse decisions made to progress ideas together, which resolve to periods of negotiation and subtle convergent work on bounded tasks. More generally, the approach taken by *Commands* was largely top-down, rather than generative and bottom-up. They divide tasks and progress through the workloads, making decisions and not backtracking unless necessary. When directly asked about their process, they highlighted that volume of work requires them to rationalise, make decisions, and progress quickly. But it is important to note that other forms of more generative work occurred in *Commands’* music making. Within the other data collected, numerous instances of embodied imagination, musical virtuosity and creative collaborative process were observed. Also, despite having a collection of external hardware drum machines, all the drum programming took place within DAW interfaces. When asked why they did not use external drum machines, Kyle responded that those devices were only really used for personal ‘artist’ projects, where the boundaries of creation are perceived as different. Given this, we wish to acknowledge that technologies designed for audio production should undoubtedly accelerate core aspects of the craft, making sounds and being creative! Further studies should assess CAP in more generative creative contexts, using a broader array of musical tools, to build a complete picture of design needs.

## Implications and Opportunities

In this section we discuss how design can be informed by our analysis of what was important to *Commands’* collaborative situation (Section 6.1). We then use these ideas to speculate on design opportunities for synchronous CAP, in co-located and remote contexts (Section 6.2).

### Audio Visual Precision and Temporal Stability

A fundamental feature observed was that *Commands* shared the same sonic and visual environment, with this they had a form of *collaborative perceptual space*. With a shared environment they were able to make precise decisions about audio production issues. This relied on a level of perceptual consistency so that suggestions were mutually understood. While a somewhat obvious feature of the face-to-face situation observed, it does have important implications for CAP system design.

Perceptual consistency is problematic as music unfolds in time. The complexity of sonic features and visual representation form a tension for design, as we must balance the needs of critical listening and the visual monitoring of information. Visual interface design for audio is challenging given the sheer amount of sources used in projects. But high levels of detail are required to allow producers to see and act on problems in different ways. This level of detail makes referencing difficult. Also, because musical objects unfold in time as sound, aspects of them are only available temporarily. This is similar to other temporal interaction resources like speech, where referencing and assessment is specific, layered, and temporally located. These factors complicate the cognitive processes involved in joint attention and mutual understanding.

Looking forward to design, sharing perceptual space and it’s consistency, brings forward technical questions about temporality in audio workspaces. Namely, are collaborators working in the same moment of audio or at different points. Something as simple as the timeline play-head presents complex issues for collaboration. Imagine two users, working in headphones, at different points in a song’s timeline. At some point, one of them requires input on a decision or issue, at that moment the other user would have sampled entirely different information before that point. This would mean common ground would not be well set out, and a phase of attunement would probably be needed. This simple example, does not even factor in more advanced groupware implementation possibilities where the entire DAW could have individual states per user, such as different looping points, individual solo/mute arrangements, and unique bypass settings on effects plugins. For synchronous work, sharing precise assessments about audio content is a central concern that groupware feature design needs to focus on and experiment with. From our findings we suggest that something like single display groupware^5^ is a logical starting point for synchronous-time CAP work, given situated awareness needs (the specific timing of gesture, shared perceptual space, and trading of temporally precise assessments).

### Design Opportunities in Audio Production Interactional Practices

We position the following design ideas within the context of the *collaborative audio production interactional processes*, set out in Section 5.2. While our findings are based on purely co-located synchronous interaction, we suggest that aspects of situated practices in the studio can generalise to certain remote system design issues. However we do not discuss asynchronous features here.

#### Support Critical Listening in a Collaborative Context

As highlighted in Section 6.1, design can explore the focused playback of sound within collaboration. Based on the negotiation of issues in Fragment II, visual inspection of DAW information requires interactional work to resolve plans. A potential solution for visual design in critical listening situations is to be able to display only certain mixer channels during certain task, similar to the image operation performed in Figure 5d, channels of importance are concatenated together for easy inspection. This could be done through macros that select mixer tracks based on tag, tick boxes, or colour codes. While current DAWs may have work-arounds for this problem already, to focus on supporting the information processing needs of CAP, making such features easy would be valuable to step away from a single user design paradigm. Also, we can leverage previous research on audio delivery mechanisms to support critical listening tasks (Fencott and Bryan-Kinns, 2012).

#### Provide Shared Interfaces for Enacting Musical Ideas

Based on the asymmetric control opportunities present in each fragment (Keir controlled the computer, Kyle did not), design should explore ways to balance content control for co-located users. A common feature of groupware design is to allow multiple input devices to control the same workspace. For CAP, this could include simple methods for changing low-level features of sound, at the individual source level or as groups of sources. In co-located settings, we can exploit research on shareable interfaces, aiming to support fluid interaction on a shared object of work (Rogers et al. 2009). Arguably, equitable access is a useful design target, rather than rigid divisions of labour. However, in opening out interfaces to equitable control using groupware, periods of pause need to be maintained, as decisions may need to be made on the stability of representations and sonic feedback, so having content that continually changes may make that difficult. This requires design to include features that ‘lock’ the state of the DAW to make assessments. More generally, the field of sound and music HCI provides an extensive exploration of collaborative musical interaction (Weinberg, 2005; Blaine and Fels, 2003). However, research often focuses on novice engagement and tightly-coupled synchronous music creation (jamming), rather than the iterative process of audio production, so further research is required at the intersection of collaboration and audio production when enacting musical ideas.

#### Provide Systems for Reviewing Musical Choices

Project and information management, over time, is an important feature of audio production as work (Sec. 4.1). Accordingly, design can explore project management systems that assist group sense-making across time-scales. Features could include: tracking the production process through digital music objects that incorporate situated metadata (McGarry et al. 2017); systems to support joint remembering (Bietti et al. 2016); providing ways to compare creative and technical decisions (Coughlan and Johnson, 2009); or, provide means of holistic version control for different sessions within a project or commission (Duignan et al. 2010).

## Conclusion

Audio producers must constantly make judgements based on audio and visual evidence while working with complex audio structures. In collaborative situations this can lead to ambiguity given a lack of stability in key information sources. To design collaborative technologies to support this problem, it is important to account tacit, interactional, interleaved practices that underpin activities in real-world settings. This study suggests that an array of human-human interaction resources are used in making aesthetic assessments. The analysis traces the centrality of making assessments in the collaborative process of doing audio production work. In music production, these assessments refine the problem space, providing a basis to share perceptual impressions of the work and speculate on further action. The findings draw attention to how the technical requirements of audio production are woven together with human interactional resources to make assessments and plan action. By viewing the studio situation through its sequential organisation, our analysis highlighted how social interaction is juxtaposed with material practices within a complex technical context.

The analysis described how digital music artefacts in-the-making allow interaction with them (perceptual), on them (control), and around them (discussion). A music artefact, in the context of the studio, creates a malleable set of resources for collaborative cognition and action. This relationship is captured within our sensitising concepts, linking how assessments of content are made on previous assessments, new perceptual information, and the possibilities of further technical control. Findings can be adapted towards the design of collaborative audio production systems emphasising the importance of embodied understanding when dealing with complex time-dependent content. Looking to design, we must ensure that targets of conversation and action maintain stable relationships allowing the process of making assessments to occur with minimal overhead. This has implications for the design of new workspaces that mediate the basic levels of human interactions. What we have uncovered in this study is a vast problem space that requires some further mapping out, through studies of this type and evaluations of new designs.

## Footnotes

1 A ghostwriter is hired to produce content that is officially credited to another person as the author. In industry, ghostwriters are often used to write songs, lyrics, and instrumental pieces.

2 Musical Instrument Digital Interface, the technical standard that describes a communication protocol, digital interface, a format to store musical information, and electrical connectors that connect a wide variety of electronic musical instruments, computers, and related music and audio devices.

3 Altering the level induces a change in frequency perception (see Fletcher and Munson’s equal loudness curve (Rumsey and McCormick, 2009)). Listening to sounds at a higher-level smooths out the variation of the audible frequency range compared to low-level listening. This also changes the perceived relationship of masking in the signal (Rumsey and McCormick, 2009).

4 Phrasing adapted from Brooker and Sharrock’s work on DAW-based collaborative audio production (Brooker and Sharrock, 2016).

5 Single shared view with simultaneous use of multiple input devices (Stewart et al. 1999).
